# Evaluating the impact of COVID-19 pandemic lockdown on education in Nigeria: Insights from teachers and students on virtual/online learning

**DOI:** 10.1186/s42269-021-00538-6

**Published:** 2021-04-20

**Authors:** Osamudiamen Ebohon, Anayochukwu Chukwunonso Obienu, Francis Irabor, Frank Iwebuke Amadin, Ehimwenma Sheena Omoregie

**Affiliations:** 1 Biochemistry Program, Department of Biological and Chemical Sciences, Faculty of Natural and Applied Sciences, Michael and Cecilia Ibru University, P.M.B. 100, Agbarha-Otor, Delta State Nigeria; 2 Department of Information Technology, Bayelsa Medical University, Yenagoa, Bayelsa State Nigeria; 3 Department of Computer Science, University of Benin, P.M.B 1154, Benin City, Nigeria; 4 Malaria Research, Molecular Biology and Toxicology Unit, Department of Biochemistry, Faculty of Life Sciences, University of Benin, P.M.B 1154, Benin City, Nigeria

**Keywords:** Nigeria, Coronavirus, Lockdown, Education, Online teaching, Pandemic, Virtual learning, COVID-19, Survey

## Abstract

**Background:**

As in many countries worldwide, due to the COVID-19 pandemic lockdown, higher institutions in Nigeria closed in March 2020 and only began reopening in October of the same year. As a result of the lockdown, some higher institutions had to quickly move from the traditional face-to-face teaching method to virtual learning. This study aimed to investigate the impact of COVID-19 lockdown on education in Nigeria and also provide recommendations that may be useful in developing remote teaching contingency strategies. Five-point Likert-scale questionnaire targeting students and teachers separately was designed to get feedbacks from both students and teachers on their experiences, issues and successes. The questionnaires were divided into five categories: virtual classrooms, course learning outcomes, alternative method of assessment, impact of online teaching and satisfaction.

**Results:**

A total of 703 students and 60 teachers from five different local universities participated in this study. All participants (> 50%) had difficulties with Internet connection. Students (67%) as well as teachers (59%) agree that they had limited interactions with one another and this negatively influence student’s satisfaction (*p* < 0.01). While students were split on the most appropriate method of assessment, teachers (63%) believe assignments and oral examinations are more suitable for online teaching. Many teachers (66%) admitted that it was difficult assessing students’ abilities and performance. Some students (> 40%) were concerned about the number of assignments given. Most teachers (84%) believe there is an increase in tendency for examination malpractice when assessment was conducted virtually. Students had significantly (*p* < 0.05) higher marks in all courses during online assessment compared to previous session involving face-to-face teaching. About 83% of teachers admitted it was difficult explaining complex scientific concepts.

**Conclusion:**

Based on the results of this study, we provided recommendations to help educational institutions in Nigeria develop remote teaching contingency strategies.

**Supplementary Information:**

The online version contains supplementary material available at 10.1186/s42269-021-00538-6.

## Background

COVID-19, a novel disease, became known when it was identified as the causative agent in reported cases of patients with pneumonia admitted in hospitals in Wuhan, China, in December 2019 (Munster et al. 2020; Zhu et al. 2020). This disease is spread through airborne zoonotic droplet, and people can get infected when in close contact with the cough and sneeze of persons who have symptoms from the virus (Kumar et al. 2020). In March 12 2020, the WHO officially declared COVID-19 also known as coronavirus a pandemic (WHO 2020). Due to this pandemic, educational institutions in most countries around the world were closed. Data from UNESCO showed that the peak in closure of schools was at the beginning of April 2020, when about 1.6 billion students were affected across 194 countries (UNESCO 2020). In March 2020, the Federal Ministry of Education in Nigeria directed the closure of all schools and they only began reopening in October, of the same year.

COVID-19 pandemic has affected higher education in Nigeria. The closure of schools meant that administrators of higher education had to come up with strategies to ensure that learning continues during the lockdown. Some Nigerian universities particularly the privately owned universities quickly moved from traditional face-to-face teaching method to remote education. As the period of total lockdown extended, more universities quickly switched to online teaching. Both the teachers and students had to adapt swiftly to the new mode of education as they were trained virtually on how to use distance learning tools. Teachers and students faced challenges in adapting to online classes and maintaining the minimal communication to support learning and development. Migrating to remote learning within a short period was difficult, especially in a developing country like Nigeria where advanced technology has not been well integrated into the educational system.

Most universities involved in online teaching provided students with learning materials and pre-recorded lecture videos. Remote teaching was done through online learning management systems such as Canvas, Zoom, Edmodo, Google Classroom and Microsoft Teams. Course materials and pre-recorded lectures were also sent to the students’ emails, uploaded to learning software and sometimes sent to students WhatsApp groups. Online teaching in Nigeria was both asynchronous and synchronous. In asynchronous learning, students can communicate and complete activities at their own time and pace, while synchronous learning activities occurred through live video and/or audio with immediate feedback (Hrastinski 2008). Management teams in these universities ensured that the quality of teaching was maintained and appropriate methods which address some of the limitations of remote teaching were used. Essays, presentations, reports, quizzes, assignments, etc. were some of the coursework-related activities adopted, while most assessments were done via virtual multiple-choice questions and oral examinations.

Several universities in Nigeria did not prepare for any contingency that may affect education such as COVID-19 pandemic lockdown; however, the management teams of some institutions were able to provide guidance and support to ensure that learning activities continued and students were assessed online. This was mostly possible because school closure occurred in an era when technological innovations and digitalization in educational context are readily available. In this study, we explored the educational challenges caused by COVID-19 in Nigeria. The authors got feedbacks from both students and teachers on their experiences, issues and successes following online teaching. The study was therefore aimed at providing basis for developing contingency strategies as well as suggesting methods that may be useful in improving online teaching in Nigerian universities.

## Methods

A cross-sectional study was carried out to explore the impact of COVID-19 lockdown on education in Nigeria. Student- and teacher-targeted questionnaires were developed using Google Forms. The questionnaires were designed to measure satisfaction using five-point Likert scale questions, in five different categories: virtual classrooms (VC), course learning outcomes (CLO), alternative method of assessment (AMA), impact of online teaching (IOT) and satisfaction (SAT). The questionnaires were outlined to recognize challenges and hurdles faced by students and teachers engaging in virtual learning. Before usage, the questionnaires were given to student and teacher volunteers and thereafter revised and amended based on their comments.

### Data collection

A shortened version of the URL to access the questionnaires developed with Google Forms was distributed to various institutions in Nigeria. The survey links were distributed to students and teachers through the University’s email and various social media platforms in the different institutions. The teacher-targeted questionnaire was distributed to faculty members irrespective of their departments, while student-targeted questionnaire was distributed amongst students in various departments and program year.

Consenting volunteer faculty members provided awarded marks when assessments were conducted virtually (online) during COVID-19 2020 lockdown and the traditional face-to-face (offline) assessment conducted in the previous session (2019) in four courses selected randomly. The names of the students as well as matriculation numbers were deleted to shield their identity.

### Exclusion criteria

All students taught and assessed virtually during the COVID-19 pandemic lockdown were included in this study. Students who were not taught and assessed online were excluded from this study. Also, teachers who did not participate in virtual teaching and assessment of students were excluded. An opening question of if students were taught and assessed virtually as well as if teachers were involved in online teaching/assessment was used to filter out students and teachers not fit to participate in this study.

### Statistical analysis

Results from the five-point Likert scale questions in both the student- and teacher-targeted questionnaires were presented in diverging stacked bar charts. Awarded marks and satisfaction grouped by program and program year were presented using box plot. Satisfaction was also demonstrated and compared on a five-point scoring system with 1 being the lowest part (very poor) and 5 the highest part (excellent) of the scale. Correlation analysis between students’ satisfaction and variables of Likert scale was done using nonparametric Spearman’s rank coefficient. Unpaired two-tailed unequal variance t-test was used to determine significant difference between marks awarded during virtual learning (2020) and traditional face-to-face teaching (2019). Data analysis was done using Tableau v3 and Statistical Package for the Social Sciences (SPSS) for windows, version 23.0 (SPSS Inc., Chicago, IL, USA).

## Results

### Demographic details

Student survey yielded 703 responses from five different universities with the highest responses coming from two universities in different states (Table 1). All undergraduate students, irrespective of program year from science- and non-science-based faculties, were targeted. Total response from science-based courses was 501, and that of non-science courses was 202. Teacher-targeted questionnaire yielded only 60 responses from five different universities (Table 1). We observed fewer responses from teachers which may be an indication that the teachers were occupied with post-teaching activities or were overwhelmed with the numerous research-based questionnaires circulating during this period of COVID-19.

**Table 1:** Demographic characteristics of participants in the student- and teacher-targeted questionnaires (n = 703)

Variable	No. of respondents (%)	
	Students	Academics
*Institution name*		
Bayelsa Medical University (Public medical university)	281 (40%)	14 (23%)
Edwin Clark University (Private university)	56 (8%)	8 (13%)
Edo University, Iyamho (Public university)	178 (25%)	8 (13%)
Michael and Cecilia Ibru University (Private university)	156 (22%)	25 (42%)
PAMO University of Medical Sciences (Private medical university)	32 (5%)	5 (8%)
*Gender*		
Male	307 (44%)	36 (60%)
Female	396 (56%)	24 (40%)
*Program category*		
Sciences	501 (71%)	49 (82%)
Non-sciences	202 (29%)	11 (18%)
*Program year*		
First year	202 (29%)	–
Second year	165 (23%)	–
Third year	178 (25%)	–
Fourth year	158 (22%)	–
*Highest academic qualification*		
O’Level	692 (98%)	–
B.Sc	11 (2%)	–
M.Sc	–	13 (19%)
Ph.D	–	47 (81%)
*Job title*		
Assistant Lecturer	–	10 (17%)
Lecturer II	–	5 (8%)
Lecturer I	–	7 (12%)
Senior Lecturer	–	21 (35%)
Associate Professor	–	10 (17%)
Professor	–	7 (12%)

### Virtual classrooms (VC)

**Fig. 1:**
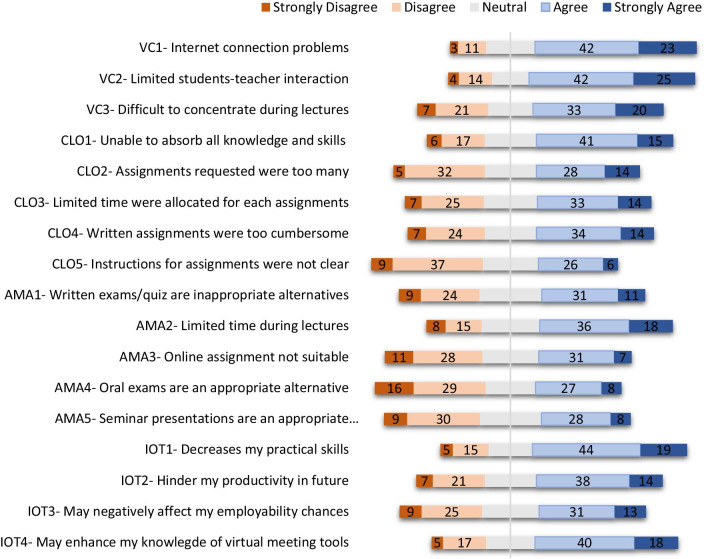
Diverging stacked bar chart showing results in percentage (%) from the five-point Likert scale student-targeted questionnaire (*n* = 703). Responses were collected in four categories: virtual classrooms (VC), course learning outcomes (CLO), alternative method of assessment (AMA) and impact of online teaching (IOT). The complete statements from the questionnaire are available in Additional file 1: Table S1

**Fig. 2:**
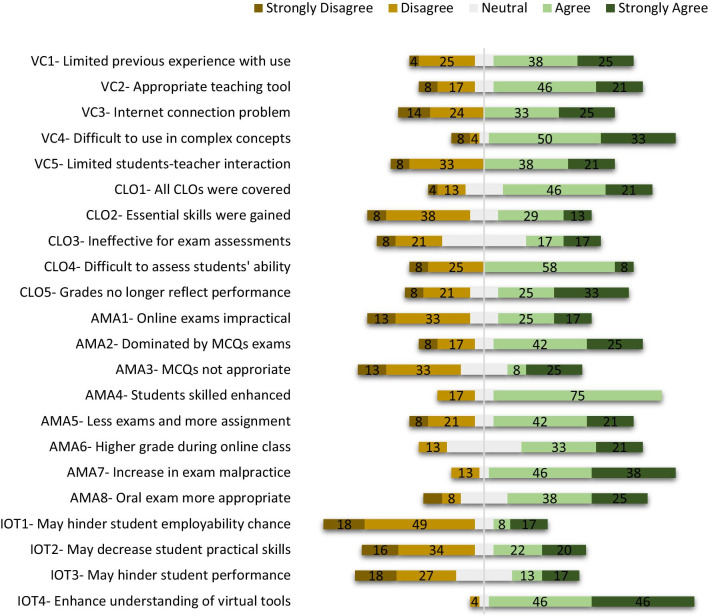
Diverging stacked bar chart showing results in percentage (%) from the five-point Likert scale teacher-targeted questionnaire (*n* = 60). Responses were collected in four categories: virtual classrooms (VC), course learning outcomes (CLO), alternative method of assessment (AMA) and impact of online teaching (IOT). The complete statements from the questionnaire are available in Additional file 1: Table S2

Result from our survey revealed that most participants (> 50%) had poor Internet connection (Figs. 1 and 2). Students (67%) and teachers (59%) agree that they had limited interactions with one another, and students claim it was one of the factors that made understanding of lecture materials difficult (Fig. 1). Approximately 53% of students claimed online teaching is not effective because of distraction (Fig. 1). Most of the teachers had limited experience in virtual teaching as only a few (29%) reported previous experience (Fig. 2). While over 60% of teachers agree that distance learning tool is effective for teaching non-practical related courses, 83% claimed it was difficult explaining complex scientific ideas or concepts to students as more time is required than traditional face-to-face teaching method (Fig. 2).

### Course learning outcomes (CLO)

More than 60% of teachers believe that all learning outcomes were completely covered except laboratory course work (Fig. 2). Teachers (46%) and students (56%) believe that essential knowledge and skills were not gained in some courses taught virtually (Figs. 1 and 2). Students raised concern on the numerous assignments given during the lockdown. Over 40% of students claimed the number of assignments was too many and limited time was allocated for each assignment (Fig. 1). Also, almost 50% of students agree that virtual assignments such as essays and reports were too cumbersome and require more efforts compared to paper-based (Fig. 1). Few students (32%) claimed that assignments instructions were not clear (Fig. 1). Approximately 34% of teachers believed distance learning tools are ineffective for examinations and over 60% admitted that it was difficult assessing students’ abilities and performance with these tools (Fig. 2). Teachers (58%) also believed that awarded grades did not reflect accurately, student’s knowledge and skills (Fig. 2).

### Alternative method of assessment (AMA)

**Fig. 3:**
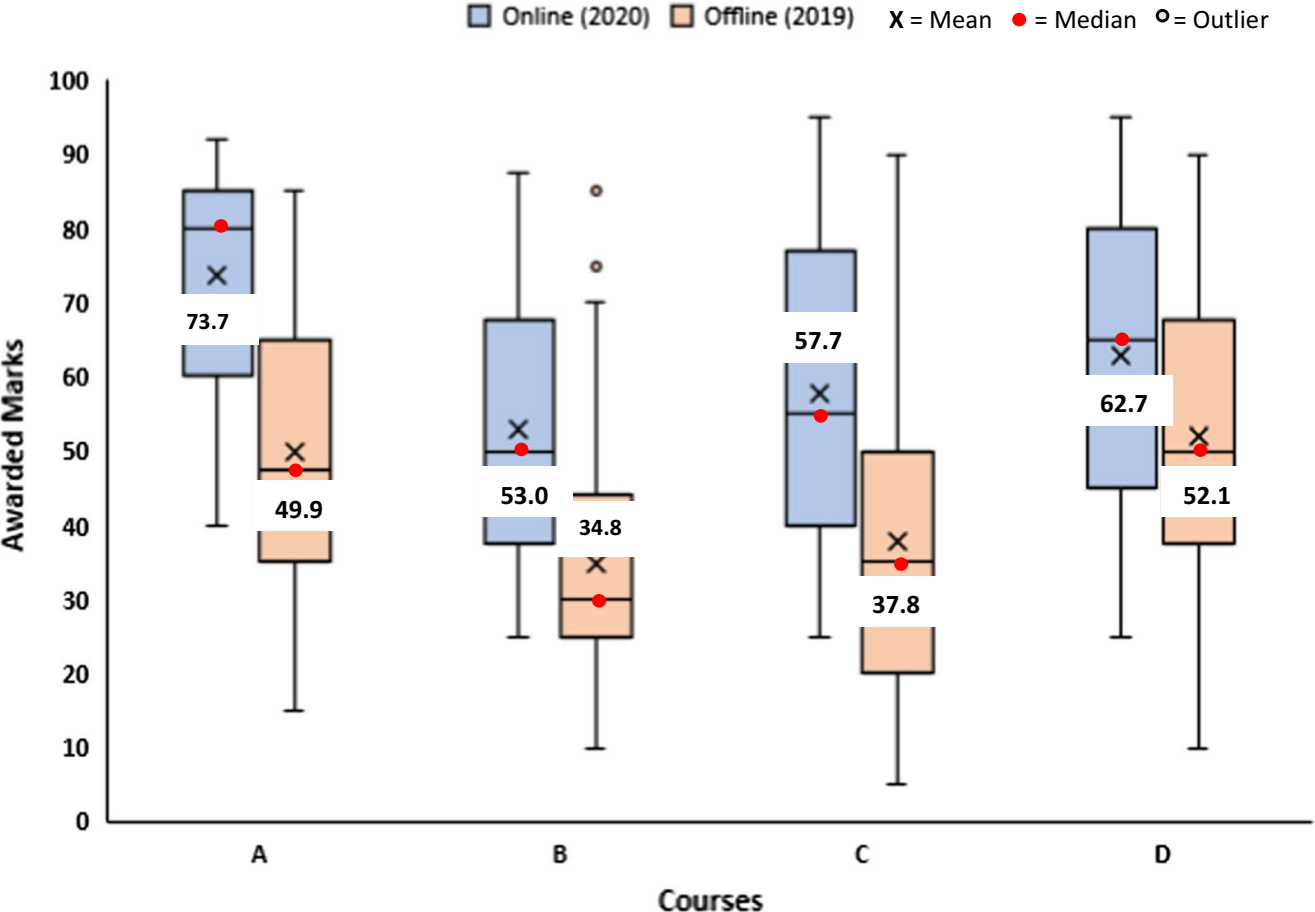
Box plot comparing marks of four courses chosen randomly from 2019 and 2020 academic sessions (no. of students per course = 206). Awarded marks in 2019 (orange colour) represent assessment done with the traditional face-to-face method (offline), while awarded marks in 2020 (blue colour) represent assessment during the COVID-19 lockdown (online). In each box, the horizontal border represents the first and third quartiles of awarded marks, upper and lower whiskers represent the highest and lowest awarded marks for each cohort, and interquartile range (IQR) rule was used to identify outliers. The awarded marks for all courses in 2020 were found to be significantly different (*p* < 0.05) from awarded marks in 2019 using an unpaired two-tailed unequal variance *t*-test

The conventional method of assessment in traditional face-to-face teaching is paper-based written examinations. However, as schools were shut during the lockdown, there was need for teachers to find alternative method of assessments. Some of the methods utilized by teachers involved assignments, multiple-choice questions, oral examinations, etc. Many teachers (75%) believe that these alternative methods of assessment enhanced student’s skills and that it should be used more often in traditional face-to-face teaching (Fig. 2). Both teachers and students (42%) agree that online examinations are unsuitable (Figs. 1 and 2). This may be because over 60% of the teachers admitted that a fair number of examinations were dominated by multiple-choice questions (MCQ) (Fig. 2). Over 50% of students claim not enough time were allocated for online written examinations, quizzes, multiple-choice questions, etc. While students were split on the most appropriate method of assessment, teacher (63%) believe assignments and oral examinations are more suitable for online teaching; however, several students were not in support of oral assessments (Figs. 1 and 2). Teachers (80%) agree that there is an increase in tendency for examination malpractice when assessments were conducted virtually (Fig. 2). Teachers (54%) claimed that students gained higher marks during online assessment when compared with traditional face-to-face teaching (Fig. 2). Interestingly, our study revealed that students gained significantly (*p* < 0.05) higher marks in all courses, when assessments were conducted virtually (online) during COVID-19 2020 lockdown as against the traditional face-to-face (offline) assessment conducted in the previous session (2019) (Fig. 3). Most of the marks awarded during online assessments were clustered mainly between 35 and 85%, while the marks awarded in the previous session (offline) were clustered between 20 and 70% (Fig. 3). The mean and median values from the box plot showing awarded marks were close and this indicates a normal distribution (Krzywinski and Altman 2014). Our findings are in agreement with teachers response on higher marks during lockdown and the claim that awarded grades may not accurately reflect student’s knowledge and skills (Fig. 2).

### Impact of online teaching (IOT)

Some teachers (42%) and students (63%) agree that virtual learning during the lockdown may decrease student’s practical skills. This is the first time that virtual learning was used fully in some Nigerian universities and some students (44%) fear that this may hinder students’ performance as a graduate and also decrease their employability chances (Fig. 1). Nevertheless, both teachers (> 80%) and students (58%) agree that online teaching may enhance their understanding of distance learning and virtual meeting tools.

### Satisfaction (SAT)

**Fig. 4:**
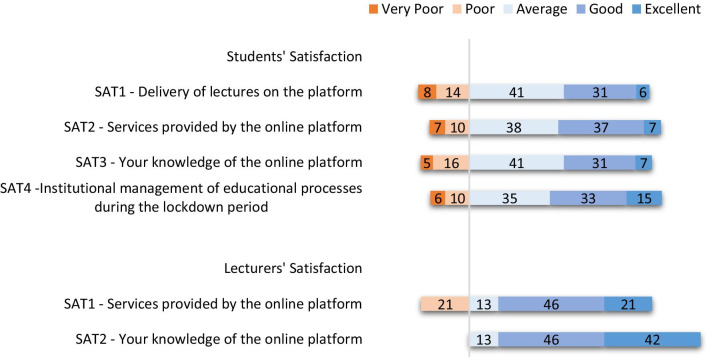
Diverging stacked bar chart showing students (*n* = 703) and teachers (*n* = 60) satisfaction (SAT) in percentage (%) from the five-point Likert-scale questionnaires. The complete statements from the questionnaire are available in Additional file 1: Tables S1 and S2

**Fig. 5:**
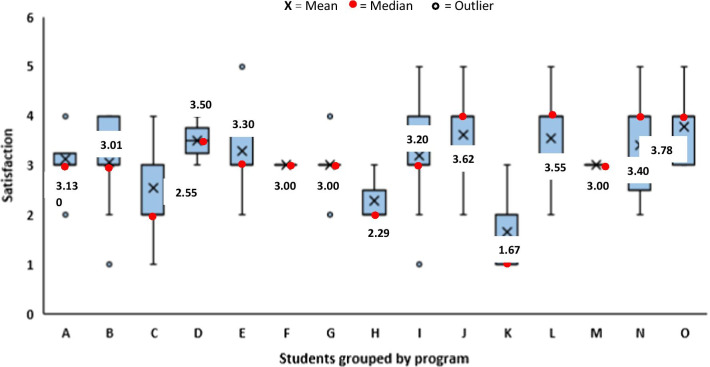
Box plot to demonstrate and compare student’s satisfaction, on a five-point scoring system, with the performance of the institution in managing educational processes during the lockdown, grouped by program. The program names were replaced with alphabets to protect their privacy. In each box, the horizontal border represents the first and third quartiles of awarded marks, upper and lower whiskers represent the highest and lowest awarded marks for each cohorts, respectively, and interquartile range (IQR) rule was used to identify outliers. The box and whiskers of program F and M were reduced to a single line because student satisfaction values were densely populated on score 3 resulting in the highest and lowest scores as well as the first and third quartiles to be the same value, where 1 = very poor, 2 = poor, 3 = average, 4 = good and 5 = excellent

**Fig. 6:**
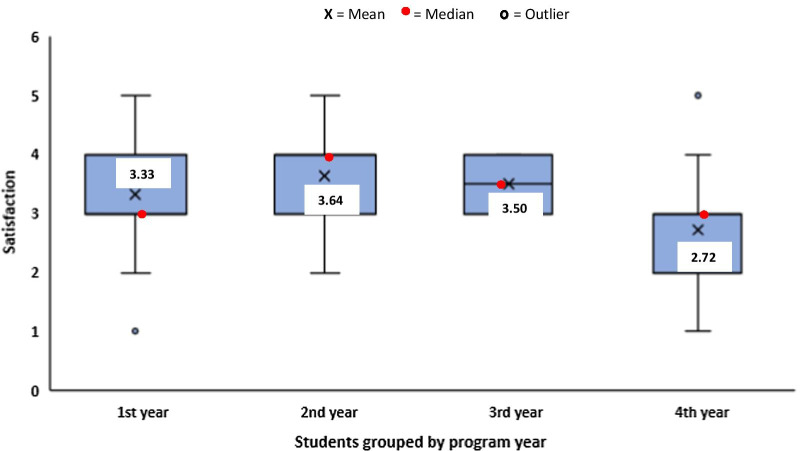
Box plot to demonstrate and compare student’s satisfaction, on a five-point scoring system, with the performance of the institution in managing educational processes during the lockdown, grouped by program year. In each box, the horizontal border represents the first and third quartiles of awarded marks, upper and lower whiskers represent the highest and lowest awarded marks for each cohorts, respectively, and interquartile range (IQR) rule was used to identify outliers, where 1 = very poor, 2 = poor, 3 = average, 4 = good and 5 = excellent

Five-point scoring system was used to express teachers’ and students’ satisfaction with the quality of the distance learning tool used (Fig. 4). Students rated their satisfaction on mode of lecture delivery and also on the way their institution managed educational process during the lockdown (Fig. 4). While most students (38%) rated the services provided by the distance learning tool as average, 37% agree it was good and 7% claimed it was excellent. On the other hand, teachers rated the services provided by the platforms used as good (46%) and excellent (21%). On the delivery of lectures on the platform, students rated it as average (41%), good (31%) and excellent (6%). Teachers had a better understanding of the distance learning tool used when compared with students. Most of the students were satisfied with the way their institution managed tutelage during the pandemic (Fig. 4). A box plot to demonstrate and compare student’s satisfaction with the performance of their institution in managing educational processes during the lockdown based on program is shown in Fig. 5. Based on the programs, most of the students were satisfied with the performance of their institutions. However, students of three departments (C, H and K), were not satisfied with their institution’s performance. A box plot to demonstrate and compare student’s satisfaction with the performance of their institution in managing educational processes during the lockdown based on program year (level) is shown in Fig. 6. We observed that students of the higher level (fourth year) were less satisfied when compared with the lower levels (Fig. 6).

We observed that the main factors that negatively influence students satisfaction significantly include limited student–teacher interaction, large number of assignments and unclear assignment instructions (*p* < 0.01) (Table 2). Other factors which do not depend on institution performance that also negatively influenced student’s satisfaction significantly are poor network connection and ease of distraction during virtual learning (*p* < 0.01) (Table 2).

**Table 2:** Correlation analysis of student’s satisfaction with the institution performance in managing educational processes during the COVID-19 lockdown and responses to questions from Fig. 1

Variable	Correlation with student satisfaction	
	*P* value	Correlation coefficient
*Virtual classrooms (VC)*		
Internet connection problems	.000	− .300**
Limited student–teacher interaction	.000	− .301**
Difficult to concentrate during lectures	.000	− .225**
*Alternative method of assessment (AMA)*		
Assignments requested were too many	.000	− .247**
Limited time were allocated for assignments	.019	− .124*
Assignment instructions were not clear	.000	− .329**

*Correlation is significant at the 0.05 level. **Correlation is significant at the 0.01 level

## Discussion

The COVID-19 pandemic lockdown affected almost every aspect of the society including everyday life. There was need for everybody to adjust to the new normal that was not pleasant and comfortable. Nigerian higher institutions were not left out in this unpleasant situation as some of these institutions had to move quickly from the traditional face-to-face teaching method to virtual learning during the lockdown. The distance learning tool used for online teaching was dependent on the choice of the institution. Many faculty members had little or no experience with virtual learning; however, management of institutions were able to quickly train these staff on tools for distance learning. Our research questions were mainly focused on getting feedbacks from students and teachers on their experiences, issues, successes, etc. following online teaching.

Result from our study revealed that faculty members of the various universities have gained knowledge and experience on distance learning tools. However, there is need for faculty members to understand all features of these tools so as to have a more interactive section with students when teaching online. One of the challenges of online teaching as noted by both students and teachers was the limited interactions they had with one another, and this limited interaction negatively affected student satisfaction significantly. Hunter et al. (2003) have previously proposed that for effective distance learning, student–student as well as student–teacher interaction is fundamental. One way to improve on the interaction between students themselves and also with their teachers is to encourage both the students and teachers to use the discussion forums in their various distance learning tools. Also, the student can create a WhatsApp group strictly for academic purposes where discussion can be done via text or voice notes. Students claimed that virtual classrooms are ineffective because they are easily distracted. Distractions are part of life, and it is the duty of the student to manage or cut off from every form of distraction. This distraction may be more prominent in students with large families or those using their smartphones for online classes. Some of the ways students can limit distraction is to attend lectures with their laptops instead of smartphones, decrease accessibility to phones during lecture hour and parents/guidance should be a source of encouragement to their children. Most teachers claim that explaining complex scientific ideas or concept to students online was difficult. In order to make complex ideas simpler, teachers can upload or post a pre-recorded detailed explanation of these concepts for students to watch before the actual virtual lecture.

A fair number of students claim they did not gain all knowledge and skills expected in some courses taught virtually. One possible reason for this claim may be due to distractions as well as limited student–student and student–teacher interactions. While students agree that the number of assignments was too many and instructions were unclear, teachers favoured more assignments and oral examinations. Result from our study showed that these perceived large number of assignments and unclear instructions negatively influenced student’s satisfaction significantly (*p* < 0.05). Although many distance learning tools provide platform for online examinations, teachers tend to have negative perception as students are not invigilated and malpractice becomes inevitable (Kaczmarczyk 2011). Indeed, our survey showed that many teachers believe that there is an increase in tendency for examination malpractice when assessment was conducted virtually. Students were not in favour of oral examination, and some of the reasons may be they usually involve wide scope, difficult to prepare for and revision is sometimes less effective (Alqurshi 2020). Our findings also showed that teachers agree it was difficult assessing student’s abilities and performance with distance learning tools and that grades were not an accurate reflection of student’s knowledge. Aside the possibility of cheating during virtual examination, one other possible reason for the teachers opinions may be most of the assessments were multiple-choice questions which are quite easy to answer. Alternative methods of assessments involving assignments, essays, reports, quizzes, oral assessments, etc. are highly encouraged. Also, problem-based learning (PBL) as part of student-centred teaching strategies is also a suitable method and it is highly encouraged in distance learning (Alqurshi 2020). Some benefits of problem-based learning are that it promotes exchange of ideas among students and encourages independence, unlike traditional face-to-face teaching where students tend to memorize lecture materials (Camp 1996). Many teachers believe that these alternative methods of assessment enhanced student’s skills and that it should be used more often in traditional face-to-face teaching, especially in courses that are assessed using multiple-choice questions. These alternative methods of assessment can easily be aligned with course learning outcome as it focuses on student’s performance and quality of work done.

Our survey revealed that teachers believe that students have gained higher marks during online assessment when compared with traditional face-to-face teaching. Indeed, teacher’s response was in agreement with our findings which revealed that students had statistically significant (*p* < 0.05) higher marks during online assessment (COVID-19 lockdown) when compared with the previous session (traditional face-to-face teaching). Our result is in agreement with Alqurshi (2020), where the author observed higher marks in all courses during COVID-19 lockdown when compared with previous sessions that were not taught virtually. The fact that during COVID-19 lockdown, a fair number of assessments were multiple-choice questions and students can sometimes find answers to questions online may be a possible explanation for the higher marks gained by students. One of the ways to manage these discrepancies in scores is to utilize more of problem-based learning which would encourage critical thinking and make it less easy to find answers online.

Some students fear that virtual learning may decrease their practical skills, hence affecting their chances of employment. It is actually expected that science students doing practical-based courses would fear loss of practical skills since they were mostly taught alternative to practical skills virtually. It is therefore encouraged that practical classes be organized post-lockdown for these set of students. Student’s chances of employment would not be affected by online lectures as distance learning using virtual learning tools has been embraced worldwide. We observed that students in their fourth year were less satisfied with the way the institution managed educational processes during the lockdown when compared with students in first to third year. One reason for this observation may be the higher level tends to be more difficult and sometimes involves more practical sessions. Generally, some of the findings from this study were in agreement with the study conducted in Saudi Arabia by Alqurshi (2020) on pharmaceutical education during the COVID-19 lockdown. Statistical analysis revealed that poor network connection influenced student’s satisfaction negatively during virtual learning. Therefore, there is need for the government and telecommunication industries to improve on network coverage and broadband services.

### Recommendations

Thankfully, pandemics are rare. The last pandemic that affected the world occurred in 1918. However, there are some events aside pandemics and diseases such as war and crisis that can result in shutting down of schools and other places of learning. One example is the Nigerian civil war (1967–1970) which had a huge impact on educational institutions. Hence, there is need for educational institutions to develop contingency strategies and methods that may be useful for emergency teaching and remote learning. These strategies may include:

1. A short course addressing the usage of online learning management systems: Zoom, Google Classroom, Edmodo, etc. should be added to the school’s curriculum.
2. The National University Commission of Nigeria should develop a document outlining student friendly teaching strategies as well as assessment methods. This document may be useful during emergency teaching.
3. Practical classes should be organized for practical-based courses post-lockdown.
4. When possible, revision session for all courses taught online should be organized and traditional assessment method utilized post-lockdown.
5. Sufficient timelines should be given for students to complete required assessments and submit assignments. These timelines should be designed based on students program and level.
6. Infrastructure for distance learning should be improved especially in public universities.
7. Most students in higher institutions in Nigeria are from humble homes and are unable to afford a laptop or smartphones, while some of them reside in regions with poor Internet connections. Therefore, the universities should lend laptops/tabs to students from the university library with clear instructions that these devices must be returned after the lockdown or pandemic crisis.
8. High-speed Internet and equipment facilities should be made available for students in their residence outside campus.
9. Network providers should improve on their Internet coverage and broadband services as well as subsidize data subscription for students and teachers.
10. Faculty members should be trained on the usage of online learning management systems for effective teaching and assessment strategies. Also, technical support should be provided for all staff.
11. Aside emails and other traditional means of communications, online learning management systems should be integrated into the normal daily teaching routine. This would facilitate students–teacher understanding of these tools.

### Study limitations and strengths

The main limitation to this study was the method of data collection. The survey aspect of this study was dependent only on students and teachers with Internet connection; hence, those living in regions without access to the Internet may have been omitted. Also, due to lack of interest, some persons involved in virtual learning may have intentionally decided not to respond to the questionnaires.

The strength of this study was on its sample size which included all students irrespective of study program and year. Also, an opening question of if participants were involved in virtual learning was used to omit those not fit for this study. Similarly, participants who did not fill their program and demographic details completely were omitted from this study.

## Conclusion

Education in Nigeria was affected greatly, during the COVID-19 lockdown. The need for novel emergency teaching methods and the switch to remote learning during the pandemic was challenging for both students and teachers. The lack of student–student and student–teacher interactions was the major limitation associated with virtual learning as against the traditional face-to-face teaching method. As most students are relatively new to virtual learning, there is need for a student-friendly guide aimed at helping students with processes as well as the methods of assessment. Teachers should develop interactive online classes to limit student’s distraction and also improve satisfaction. Nigerian government, management of higher institutions and telecommunication industries should collaborate to subsidize cost of Internet for both students and teachers. Telecommunication industries should increase Internet coverage and broadband services so as to overcome Internet-related issues. This is the first time most institutions in Nigeria are using distance learning tools and it is recommended that the Federal Government of Nigeria and the Ministry of Education should see the COVID-19 pandemic lockdown as an opportunity to invest and promote virtual learning in Nigerian institutions.

## Supplementary Information

**Additional file 1: Table S1**. The complete statements from the questionnaire.

## Data Availability

The datasets used and/or analysed during the current study are available from the corresponding author on reasonable request.

## Abbreviations

VC: Virtual classrooms
CLO: Course learning outcomes
AMA: Alternative method of assessment
IOT: Impact of online teaching
SAT: Satisfaction
