# Supplementary material for: Evaluating the impact of COVID-19 pandemic lockdown on education in Nigeria: Insights from teachers and students on virtual/online learning
Source: Bull Natl Res Cent. 2021 Apr 20;45(1):76. doi: 10.1186/s42269-021-00538-6 (PMC8057660; doi:10.1186/s42269-021-00538-6)
Supplement: Supplementary file 1 — Additional file 1: Table S1. The complete statements from the questionnaire. [file 42269_2021_538_MOESM1_ESM.docx]

**Evaluating the impact of COVID-19 pandemic lockdown on education in Nigeria: Insights from teachers and students on virtual/online learning**

**Survey on Online teaching in Nigeria during COVID-19 lockdown**

Responses from students and teachers were collected using the 5-point Likert-scale (Strongly disagree, disagree, neutral, agree, and strongly agree). Satisfaction was demonstrated and compared on a 5-point scoring system with 1 being the lowest part (very poor) and 5 the highest part (excellent) of the scale.

**Table S1:** Students-targeted Questionnaire

Demographic details of participants: Gender, program, program year, name of institution, highest academic qualification, and virtual learning tool used for learning were collected.

| **Construct** | **Item Code** | **Item** |
| --- | --- | --- |
| Virtual Classrooms (VC) | VC1 | I struggle with attending virtual classrooms due to internet connection problems. |
|  | VC2 | With remote teaching I have had limited interactions with teachers causing difficulties in understanding the lecture materials. |
|  | VC3 | Virtual classrooms are ineffective because one can be distracted easily. |
| Course Learning Outcomes (CLO) | CLO1 | During COVID-19 lockdown, I was unable to absorb all knowledge and skills associated with some of the courses and as a result I fear it may affect my performance in the future. |
|  | CLO2 | Number of assignments requested during the COVID-19 lockdown were too many. |
|  | CLO3 | Limited time were allocated for each assignments. |
|  | CLO4 | Witten assignments such as essays and reports were too cumbersome (complex) and requires a lot of effort compared to paper based. |
|  | CLO5 | During online teaching, instructions for assignments were not clear |
| Alternative Method of Assessment (AMA) | AMA1 | Online written exams or quiz are inappropriate method of assessment. |
|  | AMA2 | Enough time were not allocated for online written exams or quiz. |
|  | AMA3 | Online assignments were unsuitable method of assessment. |
|  | AMA4 | Online oral exams are better suitable for assessment during online teaching. |
|  | AMA5 | Seminar presentations are appropriate method of assessment during online lectures. |
| Impact of Online Teaching (IOT) | IOT1 | Online teaching may decrease my practical skills. |
|  | IOT2 | Online teaching may hinder my performance as a graduate of this course. |
|  | IOT3 | Online teaching may decrease my employability chances as adequate knowledge were not gained. |
|  | IOT4 | Online teaching increased my understanding of tools for virtual learning and meetings tools |
| Satisfaction (SAT) | SAT1 | How satisfied are you with the delivery of lecture on the online platform? |
|  | SAT2 | Rate the services provided by the online platform |
|  | SAT3 | Rate how well you understand the online platform |
|  | SAT4 | How satisfied are you with the way the institution managed educational processes during the lockdown period |

**Table S2:** Teacher-targeted Questionnaire

Demographic details of participants: Gender, program, name of institution, highest academic qualification, and virtual learning tool used for learning were collected.

| **Construct** | **Item Code** | **Item** |
| --- | --- | --- |
| Virtual Classrooms (VC) | VC1 | Prior to the COVID-19 quarantine phase, I had very little experience in using virtual classrooms. |
|  | VC2 | After using virtual classrooms this semester, I believe it to be an appropriate teaching tool for most courses that don’t require practicals. |
|  | VC3 | I struggle with internet connection and thus find it difficult to stay connected to a virtual classroom. |
|  | VC4 | Explaining complex scientific concepts to students through virtual classrooms is difficult and requires more time than conventional teaching. |
|  | VC5 | Using virtual classrooms resulted in limited student-teacher interaction. |
| Course Learning Outcomes (CLO) | CLO1 | During the lockdown, I believe I was able to cover all learning outcomes, except for psychomotor ones. |
|  | CLO2 | Most students have gained all required knowledge and skills; their performance in their various field in the future will not be negatively affected by the COVID-19 lockdown. |
|  | CLO3 | Distance learning tools are impractical and ineffective for exam assessments. |
|  | CLO4 | Due to COVID-19 lockdown, it was very difficult to assess students’ abilities and performance with regards to course learning outcomes using virtual learning tools. |
|  | CLO5 | Awarded grades this semester are not an accurate reflection of students’ knowledge and skills; |
| Alternative Method of Assessment (AMA) | AMA1 | Exams written online are not suitable. |
|  | AMA2 | Multiple choice questions (MCQs) exams were mainly the form of assessment. |
|  | AMA3 | Multiple choice questions (MCQs) were not an appropriate method of assessment. |
|  | AMA4 | Student skills were enhanced using alternative method of assessment. |
|  | AMA5 | Give more assignment and less exam. |
|  | AMA6 | Students had higher marks during online assessment compared to traditional face-to-face teaching. |
|  | AMA7 | There is an increase in tendency for exam malpractice |
|  | AMA8 | Oral exams are better suitable for assessment compared with assignments during online teaching |
| Impact of Online Teaching | IOT1 | Online teaching may decrease employability chances of students. |
|  | IOT2 | Online teaching may decrease practical skills of student. |
|  | IOT3 | Online teaching may hinder performance of student as a graduate of this course. |
|  | IOT4 | Online teaching would increase students understanding of tools for virtual meeting. |
| Satisfaction (SAT) | SAT1 | Rate the services provided by the online platform |
|  | SAT2 | Rate how well you understand the online platform |
